# How old is this mutation? - a study of three Ashkenazi Jewish founder mutations

**DOI:** 10.1186/1471-2156-11-39

**Published:** 2010-05-14

**Authors:** Celia MT Greenwood, Shuying Sun, Justin Veenstra, Nancy Hamel, Bethany Niell, Stephen Gruber, William D Foulkes

**Affiliations:** 1 Genetics and Genome Biology, Hospital for Sick Children Research Institute, Toronto, Ontario, Canada; 2 Dalla Lana School of Public Health, University of Toronto, Toronto, Ontario, Canada; 3 General Medical Sciences (Oncology), Case Comprehensive Cancer Center, Cleveland, Ohio, USA; 4 Department of Statistics and Actuarial Science, University of Western Ontario, London, Ontario, Canada; 5 Department of Medical Genetics, Research Institute of the McGill University Health Centre, Montreal, Quebec, Canada; 6 Diagnostic Radiology, Massachusetts General Hospital, Boston, MA, USA; 7 University of Michigan School of Public Health, Ann Arbor, Michigan, USA; 8 Cancer Genetics, Departments of Oncology and Human Genetics, Gerald Bronfman Centre for Clinical Research in Oncology, McGill University, Montreal, QC, Canada; 9 Lady Davis Institute for Medical Research, Jewish General Hospital, Montreal, QC, Canada

## Abstract

**Background:**

Several founder mutations leading to increased risk of cancer among Ashkenazi Jewish individuals have been identified, and some estimates of the age of the mutations have been published. A variety of different methods have been used previously to estimate the age of the mutations. Here three datasets containing genotype information near known founder mutations are reanalyzed in order to compare three approaches for estimating the age of a mutation. The methods are: (a) the single marker method used by Risch et al., (1995); (b) the intra-allelic coalescent model known as DMLE, and (c) the Goldgar method proposed in Neuhausen et al. (1996), and modified slightly by our group. The three mutations analyzed were MSH2*1906 G->C, APC*I1307K, and BRCA2*6174delT.

**Results:**

All methods depend on accurate estimates of inter-marker recombination rates. The modified Goldgar method allows for marker mutation as well as recombination, but requires prior estimates of the possible haplotypes carrying the mutation for each individual. It does not incorporate population growth rates. The DMLE method simultaneously estimates the haplotypes with the mutation age, and builds in the population growth rate. The single marker estimates, however, are more sensitive to the recombination rates and are unstable. Mutation age estimates based on DMLE are 16.8 generations for MSH2 (95% credible interval (13, 23)), 106 generations for I1037K (86-129), and 90 generations for 6174delT (71-114).

**Conclusions:**

For recent founder mutations where marker mutations are unlikely to have occurred, both DMLE and the Goldgar method can give good results. Caution is necessary for older mutations, especially if the effective population size may have remained small for a long period of time.

## Background

Several diseases occur at higher frequency among Ashkenazi Jewish individuals [1], and furthermore, specific mutations have been identified that are at a higher frequency in this population than in other populations around the world. For example, the mutation BRCA2*6174delT is present in approximately 1% of Ashkenazi Jews [2,3], and is present at increased frequencies in early-onset Ashkenazi Jewish breast cancer cases and families. Similarly, the mutation MSH2*1906 G->C leads to increased risk of colorectal cancer among Ashkenazi Jewish individuals [4], and the colorectal cancer-susceptibility allele APC I1307K appears to confer a relative risk of 1.5-2.0 for colorectal cancer (CRC) on carriers [5]. For each of the above three mutations, cases carry a haplotype in common surrounding the mutation site, suggesting that all cases are descendants of a recent founder. It has been argued that the high frequency of some of these mutations is due to various population bottlenecks that the population has undergone over the centuries [6,7], leading to a unique genetic signature [8]. It is, therefore, interesting to estimate the ages of these mutations in order to compare whether they arose at a similar period in the history of this population, or whether they have very different ancestries. Several quite different methods have been proposed for estimating mutation age, and hence here we evaluate and compare three approaches, and make recommendations as to when and where each method is preferable.

The simplest method for estimating mutation age, proposed by Serre et al. (1990) [9] and popularized by Risch et al. (1995) [6], is based on the expected decay of linkage disequilibrium due to recombination, between a marker and the mutation, over the generations between the origin of the mutation and today. However, consideration of gene genealogies and models for coalescent times for different chromosomal lineages can lead to richer models capable of incorporating population growth and accounting for the randomness of the gene genealogy [10]. A simple model for gene genealogies is the star genealogy where all copies of the disease-causing mutation are assumed to have one common coalescent time. Under this model, all the histories of each copy of the mutation are independent of one another; this assumption is quite reasonable for fast-growing populations where the probability of two lineages merging is small. The simple estimate (Equation (1), Additional file 1) is the maximum likelihood estimate under this model. However, the star genealogy is unlikely to be realistic. Assuming a bifurcating genealogy where all lineages split at the same time, Labuda et al. (1996) [11] showed that the estimate of Risch (1995) was likely to be biased downwards. They developed a correction factor that increases the estimated age and accounts for the population growth rate (see Additional file 1, part A).

A multiple-marker likelihood for estimating mutation age was implemented by Goldgar [12] and used to estimate the age of several mutations for BRCA1 and BRCA2 [12,13]. This likelihood-based method includes the effects of both recombination and mutation and allows for a particular mutation to occur independently more than once, but requires mutation-carrying haplotypes to be known. Different mutation rates can be set for different types of markers (e.g. dinucleotide, tetranucleotide). Population growth is not taken into account. We modified this likelihood to allow for haplotype uncertainty; all possible mutation-carrying haplotypes were included in the likelihood together with their probabilities, as estimated by PHASE, and we refer to this approach as the modified Goldgar method (Additional file 1, part C).

Rannala and Slatkin (1998) [14] used a continuous-time birth-death process to develop a likelihood for the distribution of the intra-allelic coalescent for a rare disease mutation; the generality of this model allows for the possibility of population growth and selection at the disease locus. It is also worth noting that their method estimates the time of origin of the mutation, which could be substantially older than the time of the last coalescent event. In [15], the model was extended to multipoint linkage disequilibrium mapping by calculating the probabilities of transitions between marker haplotypes, given the position of the disease locus. Markov Chain Monte Carlo methods were used to estimate the likelihood function, by sampling in sequence for tree topology and coalescent times, the haplotype on which the mutation arose, the position of the disease locus, and the ancestral haplotypes. The sampled likelihood can then be used to obtain estimates of parameters of interest. Since haplotypes are estimated, prior assumptions about phase are unnecessary. Software for fitting their multilocus model is available [16], and is known as DMLE.

### Datasets

We compared these three methods for estimating mutation age using three datasets containing cases and controls of Ashkenazi origin. Firstly, we examined 16 colorectal cancer cases carrying MSH2 1906G > C, together with 109 controls [4,17]. Among the Ashkenazi Jewish cases, two were from Canada, nine from U.S.A., one from France, two from Germany, and two were from Australia. The Ashkenazi Jewish controls were randomly sampled individuals from Montreal whose mutation status was unknown at time of sampling. None of the controls carried the mutation. Nineteen markers spanning 12.3 Mb on chromosome 2 were available for analysis, including 6 intragenic markers. Previously, it was shown that all cases carried an extended haplotype around the mutation, and furthermore that this haplotype was seen in ~10% of controls as well [4]. This dataset will be referred to as the MSH2 dataset.

Secondly, we analyzed 56 cases of colorectal cancer known to carry the APC I1307K mutation, together with 32 mutation carriers without colorectal cancer [5]. These individuals were recruited as part of a matched case-control study of incident colorectal cancer in northern Israel. In the original case-control study, matching of cases to controls was based on year of birth, gender, clinic, and Jewish or non-Jewish heritage [5] Among the 88 mutation carriers that we have analyzed, 56 were from the set of colorectal cancer cases and 32 were matched control individuals. There were three Sephardic individuals, two Arabs, and two individuals of unknown background. The remainding individuals were Askhkenazi Jewish. Six markers spanning 4.9 kb near the gene were analyzed. This dataset will be called the I1307K dataset.

Thirdly, we analyzed 34 cases of breast cancer and 63 controls [2]. Cases were Ashkenazi individuals seen in the cancer genetics service at the Memorial Sloan-Kettering Cancer Center. Twenty-three anonymous Ashkenazi Jewish controls were purchased from Israel (from the National Laboratory for the Genetics of Israeli Populations), and forty controls were randomly selected from a pool of Ashkenazi Jewish individuals in the New York area. The data were taken from a genome-wide SNP scan, but we focused on the region encompassing the BRCA2 gene. All cases were known carriers of BRCA2*6174delT; controls were unaffected by breast cancer. In a genome-wide analysis of 8487 markers, two markers were identified near BRCA2 with significantly different frequencies between cases and controls, after adjusting for multiple testing [2]. Neither of these two markers was immediately adjacent to the BRCA2 gene; one (TSC599767) was 1.75 Mb proximal, and the other (TSC1378449) was 822 kb distal. We examined several subsets of the 48 available markers spanning the mutation on chromosome 13: a set of 14 markers covering 10 Mb around the mutation and including the two markers previously identified as associated, a set of 10 markers spanning 5 Mb, and a set of 6 markers immediately adjacent to the mutation. This dataset will be called the BRCA2 dataset.

Estimates of mutation age are contrasted across the datasets and methods.

## Results

Tables 1, 2 and 3 show estimates of mutation age for the MSH2 data, the APC data, and the BRCA2 data, respectively. Single marker results were summarized by the median of the results across the available markers, and detailed results are shown in Additional files 2 and 3.

**Table 1:** Estimates of mutation age for MSH2 1906G > C^1^.

Method - Markers	Estimated mutation age (generations)	Estimated mutation age including correction for growth with r = 1.5	95% credible interval (DMLE) or 95% confidence interval (Goldgar)
Single marker results - median of 10 markers^2^	59.22	69.01	

Single marker results - median of 6 markers^3^	35.76	42.27	

DMLE with growth rate r = 1.5	N/A	16.84	[12.75, 22.45]

Goldgar method	11	17.62^3^	Without growth [6, 20] With growth [13.62, 26.62]^4^

^1 ^Estimates are from Sun et al. (2005) [17].

^2 ^Results from the 10 markers, individually, are listed in Additional file 2.

^3 ^The four markers closest to the mutation were excluded since recombination fractions cannot be accurately estimated at those distances.

^4 ^A Labuda correction of 6.62 generations was applied. This is the median Labuda correction for the six markers further from the mutation.

**Table 2:** Estimates of mutation age for APC I1307K.

Method	Estimated mutation age (generations)	Estimated mutation age including correction for growth with r = 1.125	Estimated mutation age including correction for growth with r = 1.21	95% credible interval (DMLE) or 95% confidence interval
Estimate from Niell et al. (2003)[5]	87.9 - 118			

Single marker - marker D5S135^1,3^	60.9	87.93	79.94	

Single marker - marker D5S346^2,3^	69.5	117.60	101.56	

DMLE	N/A	105.76	69.16	r = 1.21: [56.00, 83.22] r = 1.125: [85.96, 128.62]

Goldgar method^3^	28	65.57	53.55	r = 1.0: [20-39] r = 1.125: [57.57, 76.57] r = 1.21: [25.55, 64.55]

^1 ^The recombination fraction used for D5S135 was 0.0046.

^2 ^The recombination fraction used for D5S346 was 0.000385.

^3 ^Labuda growth rate corrections are used for the single marker method and the Goldgar method.

**Table 3:** Estimates of mutation age for BRCA2 6174delT.

Method	Estimated mutation age (generations)	Estimated mutation age assuming growth rate r = 1.125	Estimated mutation age assuming growth rate r = 1.21	95% credible interval (DMLE) or 95% confidence interval (Goldgar)
Neuhausen et al. (1998) [13]	29			1-LOD interval [22, 38]

Single marker results, median of 28 markers^1^	25.17	38.94	36.02	

DMLE, 6 markers	N/A	81.27	51.77	r = 1.125: [62.13, 102.34] r = 1.21: [40.53, 64.78]

DMLE, 10 markers	N/A	88.56	58.65	r = 1.125:[67.19, 113.57] r = 1.21:[45.48, 74.46]

DMLE, 14 markers	N/A	90.50	60.53	r = 1.125:[71.37, 114.24] r = 1.21:[48.49, 75.23]

Goldgar method^2^, 6 markers	9	33.31	25.42	r = 1.0: [≤ 1, 124] r = 1.125: [≤ 24.31,148.31] r = 1.21 [≤ 17.42,141.42]

Goldgar method^2^, 10 markers	17	41.31	34.42	r = 1.0: [≤ 1, 70] r = 1.125: [≤ 24.31, 94.31] r = 1.25: [≤ 17.42, 87.42]

Goldgar method^2^, 14 markers	15	39.31	32.42	r = 1.0: [≤ 1, 42] r = 1.125: [≤ 24.31, 66.31] r = 1.21: ≤ 17.42, 59.42]

^1^The single marker result shows the median estimate of mutation age across 28 out of 48 markers near the mutation for which a single marker estimate was possible (see Additional file 3). The correction for growth was based on the median Labuda correction across 28 markers for which a single marker estimate of mutation age was possible.

^2^Labuda corrections for growth are used.

For the MSH2 data, DMLE and the modified Goldgar method give very similar results of approximately 17 generations, or an estimated mutation age of 425 years, assuming 25 years per generation (Table 1). The sizes of the likelihood-based confidence interval and the credible interval also agree quite closely, estimating plausible ranges of 14 and 9.7 generations respectfully. Among the single marker results, the four markers furthest from the mutation give estimates of 12-50 generations, but the markers less than 1 cM from the mutation give very large results that are not believable.

For the I1307K data, the discrepancies between the approaches are somewhat larger (Table 2). Niell et al. (2003)[5] estimated the mutation age to be close to 100 generations, with a likely range spanning 30 generations. Assuming a growth rate of 1.125 per generation, our single marker estimates (87.9 and 117.6 generations) and the DMLE estimate (105.7 generations), are quite similar to this. However, the modified Goldgar estimate is somewhat smaller at only 65.6 generations. Altering the growth rate to 1.25 per generation leads to a substantially smaller DMLE estimate of only 69 generations. The sizes of the likely ranges around these estimates are also quite variable: the DMLE credible interval spans 42.7 generations, whereas the likelihood-based interval spans only 19 generations.

Using the original method developed by Goldgar [12], based on 10 short tandem repeat markers around BRCA2, the age of the BRCA2*6174delT mutation was estimated to be 29 generations [13]; applying a median Labuda correction for a growth rate of 1.125, this becomes 53.31 generations. Here, our estimates of mutation age depend dramatically on the method used (Table 3). Again assuming a growth rate of 1.125 generations, for 6 markers very close to the mutation, DMLE estimates 81.3 generations whereas our modified Goldgar method estimates only 33 generations. Estimates for marker sets spanning 5 Mb or 10 Mb are a little older: for the likelihood method, the estimated mutation age increases to approximately 40 generations, whereas for DMLE the estimated mutation ages are near 90 generations.

## Discussion

Estimating the age of founder mutations will always be an inexact endeavour. The true recombination and mutation history of the relevant chromosomal segments is unknown, and all models make strong assumptions that cannot be verified. Nevertheless, multi-marker approaches tend to give more consistent estimates than single marker estimates. For the latter, excellent recombination fraction estimates are crucial, and hence, estimates are particularly unreliable for markers very close to the mutation where the recombination fraction cannot be well estimated. Since the single-marker estimates are based simply on the expected decay of one haplotype, no estimates can be obtained when the allele frequency on control chromosomes exceeds the allele frequency on the mutation-carrying chromosomes.

To implement the likelihood and the single-marker methods, it is necessary to know which allele lies on the ancestral haplotype containing the mutation (the mutation-associated allele at each marker). To obtain this information, we estimated haplotypes using PHASE [18-20] and used haplotype predictions to estimate which were the associated alleles at each marker. For the single marker method, we used the most probable mutation-associated allele, but for our modification of Goldgar's likelihood method, we included all predicted mutation-carrying haplotypes, with their probabilities.

The multi-marker likelihood for one haplotype is calculated by proceeding outwards from the known disease locus, while calculating the probabilities needed to explain the data at each new marker, assuming either recombination or mutation events as required; these are then combined in a weighted sum over the possible haplotypes. Although the result can be expected to underestimate the true mutation ages since population growth is not taken into account, we corrected the estimates for growth using the median of the single-marker Labuda growth rate corrections. Although this method incorporates parameters for mutation at each marker, unlike the DMLE model, over the time frames estimated for these three data sets, the probability of marker mutation or disease locus mutation is small. For older events and tightly linked markers, however, this method should have an advantage over methods based solely on recombination.

The DMLE method estimates time to the origin of the mutation rather than to the most recent common ancestor. Furthermore, this method includes consideration of the possible variability in genealogies when calculating the posterior credible intervals. These two factors would lead one to expect wider confidence intervals and older age estimates.

Based on the DMLE estimate, the MSH2 mutation is estimated to be approximately 17 generations old, or perhaps 425 years old assuming 25 years per generation, and the likelihood-based estimate is very similar. These estimates place the origin of the mutation about in the year 1575, during a period when the community was reasonably large and undergoing fast population growth. It is very plausible that any new mutation would become rapidly and widely disseminated under these conditions [10].

In contrast, we see substantial disparities between the two multi-marker methods for the other two mutations. The I1307K mutation is estimated by DMLE to have arisen in perhaps 650 BCE (assuming r = 1.125), yet at 350 CE by the likelihood approach. Despite the large difference, these origin estimates are consistent with the fact that the mutation was seen in several Jewish populations that are not Ashkenazi, but share a common haplotype around I1307K [21]. In [14], the BRCA2 6174delT mutation was estimated to have arisen 29 generations ago, using the original version of the likelihood method. Here, our estimates, using the modified likelihood method on new marker data and without correction for population growth, were quite a bit smaller (9-17 generations), and the DMLE estimates (including corrections for growth) were substantially larger at 60 or 90 generations. In this case, the choice of method, and to a lesser extent, the choice of markers, makes a substantial difference to the result. It is worth noting, however, that the confidence intervals for the likelihood based approach are extremely wide and include the DMLE estimates and most of their credible intervals. Using DMLE, the 6174delT mutation may have arisen at approximately 200 BCE (r = 1.125) but the most recent common ancestor is estimated at 1000 CE by the Goldgar likelihood (allowing for population growth). The earlier estimate agrees perhaps better with the rare finding of the BRCA2 6174delT mutation in Sephardi Jews [22]. How can these differences be reconciled?

Since the DMLE method attempts to identify the time in history at which the mutation in question arose, rather than the most recent common ancestor, it is possible, therefore, that if a mutation were to arise in a relatively isolated Jewish community, it could be transmitted within that community for a substantial number of generations before branching out into ostensibly unrelated Jewish individuals. In this situation, the most recent common ancestor could be considerably closer to the current time than is the origin of the mutation. This phenomenon, while entirely speculative, could explain the data obtained here, wherein DMLE estimates for both the APC and BRCA2 mutation place the origin of these mutations considerably further back in time than do the other methods. While it seems unlikely that the origin and wide dissemination of a mutation could be separated by more than 1000 years, we do not know in what place or Jewish sub-group this mutation arose, and prolonged geographical or cultural isolation could potentially lead to such an effect.

Also, the DMLE results change quite a lot when the growth rate is altered; it appears that a slower growth rate leads to much more potential variability in genealogies in this data set. We used simple estimates of recombination rates based on physical distance, and hence results based on the 6 closest markers may be less accurate than those based on markers spanning a longer distance.

The accuracy of any method will be only as good as the accuracy of the required parameters. Over short time scales (in evolutionary terms) such as those observed in all three of our data sets, mutation rates will be hard to estimate, and over short physical distances, recombination rates will be imprecise. Assuming that the chromosomal region of interest is well-behaved, the optimal set of markers to use when estimating age would include markers far enough away that the recombination fraction can be adequately estimated, yet not so far that the associated haplotype is no longer identifiable. Furthermore, the growth rate of the population has a very large impact on the estimated age of the mutations. Over these time periods and for the Jewish population of these three data sets, some estimates of population size are available. However, the assumption of a constant growth rate over time, which is used in both the Labuda correction and in DMLE, is known to be untrue. The Ashkenazi population experienced distinct periods of fast population growth and subsequent population bottlenecks [7], and these events are not taken into account in the estimation of mutation age. Nevertheless, since both DMLE and the Labuda correction assume constant growth rates, our comparison of methods is still interpretable.

Some other approaches for estimating mutation age have been proposed. One method is based primarily on the rate of occurrence of mutations over time, near the locus of interest [23]. This approach, which is implemented in the software BATWING, assumes no recombination, and hence is ideally suited to very small regions of the genome, or to mitochondrial DNA where there is no recombination. Since mutations occur quite rarely, this approach is particularly suited to estimating events that occurred a long time ago. A more recent approach [24] examines haplotype sharing between individuals, and constructs a phylogenetic tree from the similarity matrix. This approach is less dependent on linkage disequilibrium, but does not take the possible variability in the genealogies into account.

## Conclusions

Knowing the probable age of a mutation is primarily of academic interest, however, it does provide some insights into demography and history in special populations. Several different models have been proposed for estimating mutation age. We have demonstrated here that single marker, likelihood-based, or coalescent-based methods are likely to agree well when a mutation is young and the population is fast-growing. However, for older mutations, different methods will give more variable results. If some of the markers are likely to mutate, then our modified Goldgar likelihood is a good choice. However, in general, the coalescent model implemented in DMLE is probably the best representation of the gene genealogies and their variability. This has been recently demonstrated in a study where the date of origin of a recent mutation was known, and the DMLE estimate was very accurate [25].

## Methods

Population allele frequencies were estimated by allele counting in control individuals. When haplotypes were required, we used the PHASE algorithm [18-20] to estimate the distribution of possible haplotypes for each individual. Mutation-carrying haplotypes were then selected from these distributions, together with their probabilities.

Population growth rates were estimated using formula; p_1 _= p_0 _* r^g ^. Here, g is the number of generations, p_1 _and p_0 _are the population sizes at different times, and the growth rate is e^r ^-fold per generation_. _From [6], in the year 1900, there were 5,000,000 Ashkenazi Jewish individuals, and there were 560,000 in the year 1765. Therefore the growth rate was approximately 1.5-fold per generation (assuming 25 years per generation) over this period. This fast growth likely started near the year 1500, when there were estimated to be approximately 11,000 Jewish people in a north-eastern European region; again the growth rate up to the year 1900 can be estimated to be near 1.5. However, prior to the year 1500 there are little data on the size of the Jewish population, which probably fluctuated but remained small. We assumed lower growth rate estimates of 1.125 and 1.21 for analyses of older mutations. The former figure was obtained by assuming the population was near 11,000 in the year 600, and grew to 5 million in the year 1900. The latter figure was obtained under the same assumptions but assuming the population was 11,000 in the year 1100. Niell et al. (2003) [5] also used an estimated growth rate of 1.125-fold per generation, although they assumed this growth rate applied between 200BC and AD 0.

Single marker estimates of mutation age assumed that recombination rates were approximately 1 cM/Mb, and used the formulae in Additional file 1, part A. Estimates of mutation age using the DMLE software assumed that the affected individuals' chromosomes carry the disease mutation and that the population from which these chromosomes are sampled follows an exponential growth rate. Another key parameter in DMLE is the proportion of the population sampled; Additional file 1, part B gives details of this calculation for the three datasets.

The modified likelihood method obtains the maximum likelihood estimates for the number of generations by direct search. Hence, all our estimated ages are given as integers, since we searched for the maximizing parameter values over a grid of size one generation. 95% confidence intervals were estimated from log-likelihood differences. No correction for population growth is included in this likelihood, so we then added a simple correction for population growth to the results (adding the same correction to the boundaries of the confidence intervals), using the Labuda formula in Additional file 1, part A.

It is not possible to calculate estimates of mutation age using all 48 markers available for the BRCA2 dataset: computations become too lengthy, the region spanned is large, and it is impossible to identify an ancestral haplotype. Therefore, we selected three subsets of markers and compared the results across methods and across marker subsets. The first subset contained 15 markers, consisting of approximately every third marker from the set of 48, and specifically including the two markers previously shown to be associated [2]. This subset spans 10 Mb. Our second subset contains 10 markers, spanning only about 5 Mb, and including one of the two previously associated markers. Finally, we chose a set of 6 markers immediately adjacent to the mutation; this set also included one of the previously associated markers (see Additional file 3 for details of which markers were used).

## Authors' contributions

NH managed the Montreal dataset and generated some of the data; SS and JV performed most of the statistical analyses. SG provided the data on the I1307K mutation, and BN provided assistance with understanding these data. CG supervised the entire project, performed some of the analysis, and wrote the manuscript, with contributions from WF, who proposed the project and provided the MSH2 data. The final version of the manuscript was approved by all authors.

## Supplementary Material

Additional file 1 **Appendix describing statistical methods**. There are three sections in Additional file 1. Part A. Details on the single marker methods for estimating mutation age. Part B. DMLE parameter settings. Part C. Goldgar likelihood and modifications. Click here for file

Additional file 2 Single marker results for MSH2*1906 G → C mutation. Click here for file

Additional file 3 **Single marker calculations for the BRCA2 data set, and identifying which markers were used in the multi-marker calculations**. Single marker calculations for the BRCA2 data set for all 48 markers. Three different subsets of markers were used in multi-marker calculations, and these subsets are identified. Click here for file
