# Supplementary material for: How old is this mutation? - a study of three Ashkenazi Jewish founder mutations
Source: BMC Genet. 2010 May 14;11:39. doi: 10.1186/1471-2156-11-39 (PMC2889843; doi:10.1186/1471-2156-11-39)
Supplement: Additional file 1 — Appendix describing statistical methods. There are three sections in Additional file 1. Part A. Details on the single marker methods for estimating mutation age. Part B. DMLE parameter settings. Part C. Goldgar likelihood and modifications. [file 1471-2156-11-39-S1.DOC]

**Additional File 1.**

**Appendix describing statistical methods.**

1. **Details on the single marker methods for estimating mutation age.**

A single marker estimate of mutation age can be obtained from the following formula [6]:

$\hat{g}$**=** $\frac{\ln(\delta)}{\ln(1-\theta)}$ (1)

Here "$\theta$" is the recombination rate between the specific marker locus and the mutation locus, and δ is the linkage disequilibrium, $\delta$=(Pd-Pn)/(1-Pn). The quantities Pd and Pn are the frequencies of the the associated allele on mutation-bearing and normal chromosomes respectively.

The Labuda modification [11] adds – (1/r) ln[$\theta$ e^r^/( e^r^ -1)] to the estimate obtained from equation (1) to account for population growth.

1. **DMLE parameter settings**

Proportion of population sampled

For MSH2 data [17], the proportion of mutation carrying chromosomes sampled was estimated from a lifetime risk of colorectal cancer of 6.85%, a worldwide population of Ashkenazi Jews of 13 million and mutation prevalence among colorectal cancer cases of 0.59%. This leads to about 5254 mutation carrying chromosomes in the population, we have sampled 16 cases. So the desired proportion is 0.003045 (16/5254).

For the I1037K data, proportion of population sampled is 0.0001185372 = 94/793,000. This estimate is based on the assumption that 6.1% of the AJ population carries the I1307K mutation [5], and hence 13 million x 6.1% is 793,000. There were 89 cases identified, five of whom were homozygous for the mutation.

For the BRCA2 data, Ashkenazi Jewish population is 13 million, and approximately 1% of the AJ population carries the BRCA2 mutation [2]. Thirty-four cases were studied, so the proportion of the mutation-carrying chromosomes sampled is estimated to be 0.0002615385 = 34/130,000.

Other DMLE parameter settings remained at their default values.

1. **Goldgar likelihood and modification**

Let N be the number of cases carrying a specific mutation, and let *h_i_=1,…H_i_* denote the possible haplotypes at markers near the mutation for person i. When haplotypes are not observed, they can be estimated, but the estimation process identifies a set of haplotypes that are possible given the genotype data, and each possible haplotype will be associated with a probability *p_hi_* of being *,* the true haplotype. For simplicity, only haplotypes with non-zero probabilities are included in the set of possibilities. The method proposed by Neuhausen et al. (1996) [12] for estimating mutation age assumed that haplotypes were known. When they are unknown, the likelihood can be written as

$Lik=\prod_{i=1}^{N} \left[\sum_{h_{i}=1}^{H_{i}} p_{h_{i}}Lik_{i}\right]$

where the individual likelihood *Lik_i_* is a simplification of the one given in the 1996 paper, namely,

$$Lik_{i}=\{\mu_{D}\prod_{l=1}^{L} p(a_{hl})+(1-\mu_{D})L_{h}(1)\prod_{l=1}^{L-1} \frac{L_{h}(l,l+1)}{L_{h}(l)}\}.$$

In the second expression, $\mu_{D}$ is the probability of an independent, identical mutation at the mutation gene. If the mutation occurred again independently, then the likelihood of observing a particular haplotype is just the product of the control allele frequencies $p(a_{hl})$for allele *a_hl_* in the *h^th^* possible derivative haplotype and for the *l^th^* locus.

If the mutation is ancestral, then the likelihood depends on the probabilities of recombination and mutation at the markers. $L_{h}(l)$ is the probability of having alleles that match or do not match the ancestral alleles at locus *l*, and *L_h_(l,l+1)* is the probability of matches or mismatches at two adjacent loci. These expressions follow the notation in [12]. We allowed for SNP markers by allowing a different (smaller) mutation rate for such markers. The ancestral haplotype was chosen to be the most commonly observed haplotype among chromosomes carrying the mutation.
