# Supplementary material for: How old is this mutation? - a study of three Ashkenazi Jewish founder mutations
Source: BMC Genet. 2010 May 14;11:39. doi: 10.1186/1471-2156-11-39 (PMC2889843; doi:10.1186/1471-2156-11-39)
Supplement: Additional file 2 — Single marker results for MSH2*1906 G → C mutation. [file 1471-2156-11-39-S2.DOC]

**Additional file 2.**

**Single marker results for MSH2*1906 G🡪 C mutation^1^.**

| **Markers** | **Recombination rate between marker and mutation** | **Estimated mutation age**  **(generations)** | **Labuda correction, r=1.5** | **Estimated age including Labuda correction** |
| --- | --- | --- | --- | --- |
| D2S2331 | 0.077 | 13.25 | 3.61 | 16.86 |
| D2S2306 | 0.045 | 7.18 | 4.93 | 12.11 |
| D2S391 | 0.011 | 42.40 | 8.31 | 50.71 |
| Rs1374749 | 0.0098 | 67.33 | 8.69 | 76.02 |
| Rs91983 | 0.0040 | 51.11 | 10.89 | 62.00 |
| D2S2227 | 0.00039 | 723.03 | 16.67 | 739.70 |
| CA3 | 0.00088 | 256.79 | 14.65 | 271.44 |
| CA1 | 0.00090 | 256.33 | 14.59 | 270.92 |
| CA2 | 0.0011 | 136.55 | 14.10 | 150.65 |
| D2S123 | 0.049 | 29.11 | 4.71 | 33.82 |

^1^Results are taken from Table 1 in Sun et al. 2005 [17].
