# Supplementary material for: How old is this mutation? - a study of three Ashkenazi Jewish founder mutations
Source: BMC Genet. 2010 May 14;11:39. doi: 10.1186/1471-2156-11-39 (PMC2889843; doi:10.1186/1471-2156-11-39)
Supplement: Additional file 3 — Single marker calculations for the BRCA2 data set, and identifying which markers were used in the multi-marker calculations. Single marker calculations for the BRCA2 data set for all 48 markers. Three different subsets of markers were used in multi-marker calculations, and these subsets are identified. [file 1471-2156-11-39-S3.DOC]

**Additional file 3.**

**Single marker calculations for the BRCA2 data set, identifying which markers were used in the multi-marker calculations.**

| **Marker (TSC-ID)** | **Physical distance** | **Assumed Ancestral allele^1^** | **Recombin-ation fraction**^2^ | **Pd**^3^ | **Pn**^3^ | **Genera-tions** | **Labuda**  **r=1.125** | **Labuda**  **r=1.21** | **Labuda r=1.5** | **6-marker analysis** | **10-marker analysis** | **14-marker analysis** |
| --- | --- | --- | --- | --- | --- | --- | --- | --- | --- | --- | --- | --- |
| 987224 | 25269907 | 1 | 0.056 | 0.731 | 0.873 | NA | 5.806 | 5.927 | 4.396 |  |  | √ |
| 273115 | 25720367 | 1 | 0.052 | 0.946 | 1 | NA | 6.508 | 6.361 | 4.6 |  |  |  |
| 606788 | 25886242 | 1 | 0.05 | 0.816 | 0.981 | NA | 6.783 | 6.531 | 4.68 |  |  |  |
| 597395 | 26215881 | 2 | 0.047 | 0.612 | 0.435 | 24.261 | 7.357 | 6.885 | 4.846 |  |  | √ |
| 810730 | 26330934 | 2 | 0.046 | 0.757 | 0.576 | 18.249 | 7.566 | 7.015 | 4.907 |  |  |  |
| 128714 | 27040584 | 1 | 0.039 | 0.659 | 0.952 | NA | 8.992 | 7.896 | 5.322 |  |  |  |
| 56508 | 27352428 | 2 | 0.035 | 0.84 | 0.55 | 12.181 | 9.705 | 8.336 | 5.529 |  |  | √ |
| 1101985 | 27580400 | 2 | 0.033 | 0.966 | 0.922 | 16.979 | 10.267 | 8.683 | 5.692 |  |  |  |
| 609645 | 27836934 | 2 | 0.031 | 0.504 | 0.19 | 30.49 | 10.948 | 9.104 | 5.89 |  |  |  |
| 1587575 | 27923076 | 1 | 0.03 | 0.645 | 0.765 | NA | 11.189 | 9.253 | 5.96 |  |  | √ |
| 1587576 | 27923197 | 2 | 0.03 | 0.646 | 0.375 | 27.675 | 11.19 | 9.253 | 5.96 |  |  |  |
| 1587577 | 27923222 | 2 | 0.03 | 0.645 | 0.317 | 24.292 | 11.19 | 9.253 | 5.96 |  |  |  |
| 66511 | 28009707 | 2 | 0.029 | 0.678 | 0.397 | 26.055 | 11.439 | 9.408 | 6.032 |  |  | √ |
| 598727 | 28436292 | 2 | 0.025 | 0.905 | 0.788 | 23.838 | 12.792 | 10.244 | 6.425 |  |  |  |
| 56402 | 28657148 | 2 | 0.022 | 0.888 | 0.672 | 18.42 | 13.588 | 10.735 | 6.657 |  |  |  |
| **599767^4^** | 28683624 | 2 | 0.022 | 0.769 | 0.857 | NA | 13.688 | 10.798 | 6.686 |  | √ | √ |
| 599769 | 28683929 | 2 | 0.022 | 0.988 | 0.843 | 3.549 | 13.69 | 10.798 | 6.686 |  |  |  |
| 42060 | 28723354 | 2 | 0.022 | 0.8 | 0.585 | 29.888 | 13.842 | 10.892 | 6.73 |  |  |  |
| 42059 | 28723454 | 1 | 0.022 | 0.998 | 1 | NA | 13.842 | 10.892 | 6.731 |  |  |  |
| 820373 | 29113117 | 1 | 0.018 | 0.525 | 0.567 | NA | 15.514 | 11.926 | 7.216 |  |  |  |
| 605013 | 29417092 | 2 | 0.015 | 0.965 | 0.459 | 4.475 | 17.095 | 12.903 | 7.676 |  | √ |  |
| 56993 | 29468800 | 1 | 0.014 | 0.916 | 0.981 | NA | 17.396 | 13.089 | 7.763 |  |  | √ |
| 56992 | 29468847 | 2 | 0.014 | 0.705 | 0.177 | 30.778 | 17.397 | 13.089 | 7.763 |  | √ |  |
| 820733 | 29505201 | 1 | 0.014 | 0.834 | 0.929 | NA | 17.615 | 13.224 | 7.826 |  |  |  |
| 54081 | 29703053 | 1 | 0.012 | 0.858 | 0.948 | NA | 18.912 | 14.025 | 8.203 |  | √ | √ |
| 41500 | 29731340 | 2 | 0.012 | 0.891 | 0.597 | 26.808 | 19.115 | 14.15 | 8.262 |  |  |  |
| 208530 | 29892055 | 1 | 0.01 | 0.995 | 1 | NA | 20.369 | 14.926 | 8.627 |  |  |  |
| 1083028 | 29892548 | 2 | 0.01 | 0.903 | 0.639 | 30.878 | 20.373 | 14.928 | 8.628 |  | √ | √ |
| 195208 | 29892747 | 1 | 0.01 | 0.904 | 0.946 | NA | 20.375 | 14.929 | 8.628 | √ |  |  |
| 599568 | 29894347 | 2 | 0.01 | 0.931 | 0.732 | 29.426 | 20.389 | 14.937 | 8.632 | √ |  |  |
| 815459 | 30002886 | 2 | 0.009 | 0.934 | 0.73 | 31.072 | 21.357 | 15.536 | 8.913 | √ |  |  |
| **Mutation** | **30901018** |  | **0.0** |  |  |  |  |  |  | √ | √ | √ |
| 991493 | 31283404 | 1 | 0.004 | 0.969 | 0.737 | 32.736 | 28.606 | 20.015 | 11.019 | √ |  |  |
| 982693 | 31341701 | 1 | 0.004 | 0.935 | 0.85 | 128.606 | 27.401 | 19.271 | 10.669 | √ |  |  |
| **1378449^4^** | 31468145 | 2 | 0.006 | 0.846 | 0.823 | 358.818 | 25.26 | 17.947 | 10.047 | √ | √ | √ |
| 64502 | 31729699 | 2 | 0.008 | 0.997 | 0.952 | 7.757 | 22.04 | 15.958 | 9.112 |  |  |  |
| 983347 | 31881024 | 1 | 0.01 | 0.757 | 0.967 | NA | 20.616 | 15.078 | 8.698 |  | √ |  |
| 904475 | 31972571 | 2 | 0.011 | 0.918 | 0.935 | NA | 19.858 | 14.61 | 8.478 |  |  | √ |
| 905226 | 32023415 | 2 | 0.011 | 0.73 | 0.382 | 50.887 | 19.465 | 14.367 | 8.364 |  | √ |  |
| 43705 | 32658437 | 2 | 0.018 | 0.918 | 0.565 | 11.786 | 15.66 | 12.016 | 7.259 |  | √ |  |
| 96302 | 32703887 | 1 | 0.018 | 0.866 | 0.857 | 152.081 | 15.444 | 11.882 | 7.196 |  |  | √ |
| 51089 | 33791355 | 1 | 0.029 | 0.916 | 0.984 | NA | 11.442 | 9.409 | 6.033 |  | √ |  |
| 985640 | 34364357 | 2 | 0.035 | 0.634 | 0.15 | 16.003 | 9.911 | 8.463 | 5.588 |  |  |  |
| 149793 | 34364427 | 1 | 0.035 | 0.685 | 0.933 | NA | 9.911 | 8.463 | 5.588 |  |  | √ |
| 618894 | 34498510 | 1 | 0.036 | 0.657 | 0.967 | NA | 9.589 | 8.265 | 5.495 |  |  |  |
| 466138 | 34567529 | 2 | 0.037 | 0.66 | 0.661 | NA | 9.428 | 8.165 | 5.448 |  |  |  |
| 978933 | 34725917 | 2 | 0.038 | 0.833 | 0.596 | 13.703 | 9.071 | 7.944 | 5.344 |  |  | √ |
| 583083 | 35148937 | 2 | 0.042 | 0.806 | 0.632 | 17.298 | 8.184 | 7.396 | 5.087 |  |  |  |
| 806417 | 35931280 | 1 | 0.05 | 0.509 | 0.719 | NA | 6.757 | 6.514 | 4.672 |  |  |  |

^1^The ancestral allele was assumed to be the one more common on case haplotypes.

^2^Recombination fractions were estimated from physical distances.

^3^ ‘Pd’ and ‘Pn’ are the estimated frequencies of the assumed ancestral allele among cases and controls, respectively. The number of generations cannot be

estimated when Pd is smaller than Pn.

^4^These two markers showed association in the genome-wide SNP analysis of Ellis et al. (2006) [2].
